# Stem Cell Ribonomics: RNA-Binding Proteins and Gene Networks in Stem Cell Differentiation

**DOI:** 10.3389/fmolb.2015.00074

**Published:** 2015-12-22

**Authors:** Patrícia Shigunov, Bruno Dallagiovanna

**Affiliations:** Stem Cells Basic Biology Laboratory, Carlos Chagas Institute, Oswaldo Cruz FoundationCuritiba, Brazil

**Keywords:** ribonomics, RNA-binding proteins, stem cells, gene network, differentiation

## Abstract

Stem cells are undifferentiated cells with the ability to self-renew and the potential to differentiate into all body cell types. Stem cells follow a developmental genetic program and are able to respond to alterations in the environment through various signaling pathways. The mechanisms that control these processes involve the activation of transcription followed by a series of post-transcriptional events. These post-transcriptional steps are mediated by the interaction of RNA-binding proteins (RBPs) with defined subpopulations of RNAs creating a regulatory gene network. Characterizing these RNA-protein networks is essential to understanding the regulatory mechanisms underlying the control of stem cell fate. Ribonomics is the combination of classical biochemical purification protocols with the high-throughput identification of transcripts applied to the functional characterization of RNA-protein complexes. Here, we describe the different approaches that can be used in a ribonomic approach and how they have contributed to understanding the function of several RBPs with central roles in stem cell biology.

## Introduction

Stem cells are undifferentiated cells that are found in multicellular organisms both throughout embryonic development and in adult tissues. These cells have the ability to self-renew and the potential to differentiate into all body cell types. This plasticity makes stem cells especially attractive for use in cell therapies.

Embryonic stem cells respond to and follow a developmental genetic program that is triggered by a complex cascade of regulatory molecules. Adult stem cells remain in small amounts in adult tissues, where they are responsible for tissue repair and homeostasis (Zummo et al., 2007). Adult stem cells are able to perceive the environment through various signaling pathways that are activated by extracellular molecules and respond to these stimuli by changing their quiescent state via the activation of proliferation or differentiation (Dalton, 2013; Watt and Huck, 2013). The mechanisms that control these traits in embryonic and adult stem cells involve several steps of regulation, starting with the activation of transcription, followed by a series of post-transcriptional events (Cassar and Stanford, 2012; Cheung and Rando, 2013; Christie et al., 2013). RNA-binding proteins (RBPs) are essential mediators in post-transcriptional regulation. Interaction of RBPs with mRNAs result in complex genetic networks, and their characterization is essential to understand stem cell commitment. Here, we describe the current scenario of RNA-protein networks in stem cells and the different ribonomic approaches used in their identification.

## Stem cells and the post-transcriptional regulation of gene expression

The importance of post-transcriptional regulation has been gaining prominence since it was demonstrated that, in most cases, the transcriptome does not correlate with the proteome. This comparison contributed to the significance of post-transcriptional and translational regulation in the control of protein expression (Futcher et al., 1999; Gygi et al., 1999; Tenenbaum et al., 2000; Jayaseelan et al., 2014). In eukaryotes, transcription occurs in the nucleus, and mRNAs are translated in the cytoplasm. This spatial localization allows several sequential steps of regulation in order to achieve a fine-tuning regulation of the fate of cellular mRNAs (Glisovic et al., 2008). The desired transcript expression is mediated by different trans-acting regulators, such as RBPs and regulatory non-coding RNAs, which are organized in ribonucleoprotein complexes (RNPs). RBPs influence the structure and interactions of mRNAs and play critical roles in their biogenesis, stability, function, transport, and cellular localization (Lunde et al., 2007).

The diversity of RBPs allows cells to use them in an enormous array of combinations, giving rise to a unique RNP for each mRNA (MacKereth and Sattler, 2012). The orchestration of different RNPs in response to various stimuli prompted the concept of the RNA Regulon (Keene, 2007).

Technological advances have enabled the development of several strategies to identify and characterize RBPs and the RNAs with which they interact. In recent years, the Ribonomic approach has been applied to the functional characterization of RNPs in a wide range of eukaryotic model organisms. Ribonomics is defined by the combination of classical biochemical purification protocols with the high-throughput identification of transcripts (Tenenbaum et al., 2002).

**Figure 1:**
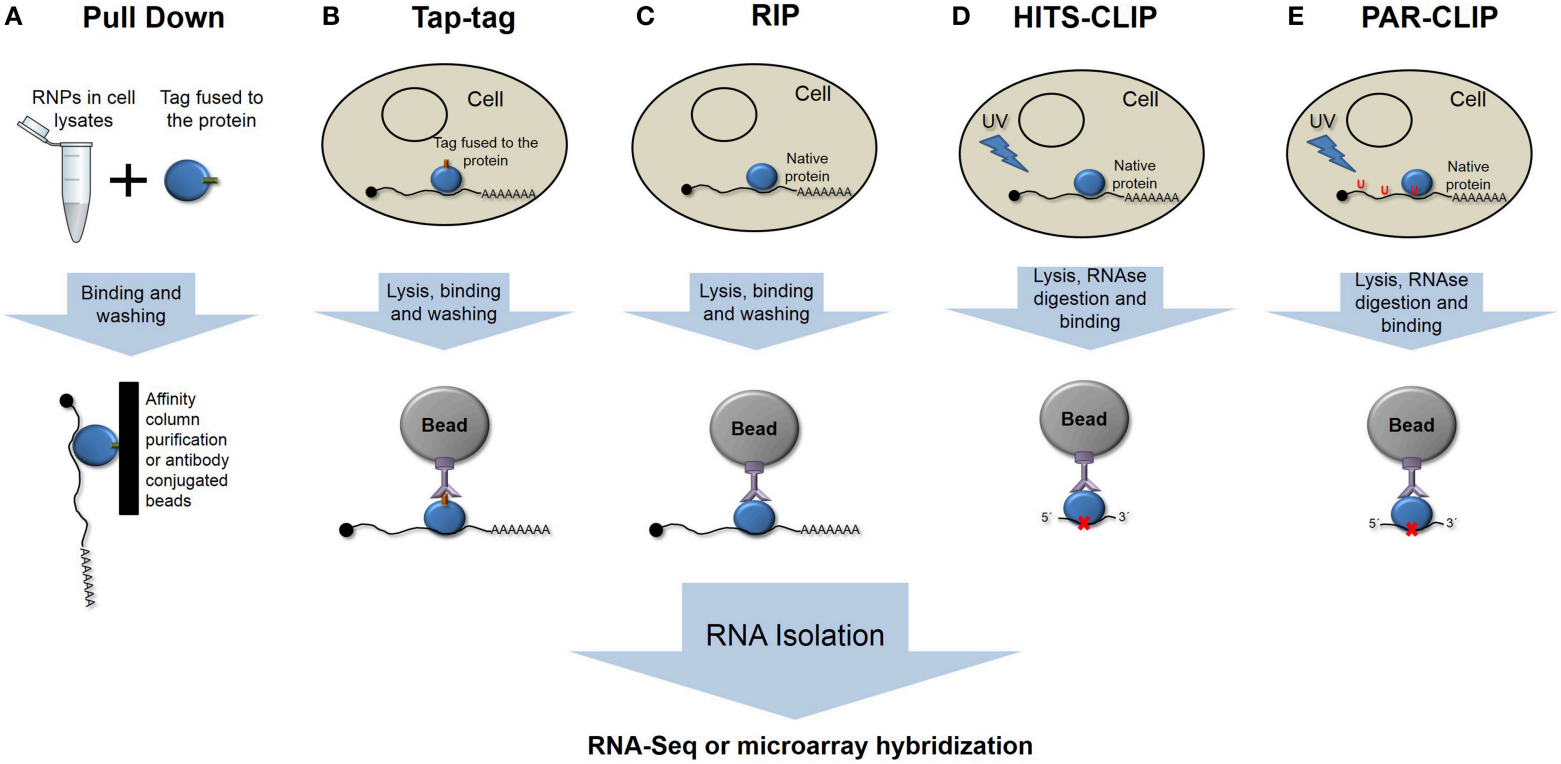
**Schematic representation of the different main ribonomic strategies. (A)** RNA pulldown *in vitro* purification. **(B)** Tandem affinity purification of tagged proteins. **(C)** RNA-protein immunoprecipitation. **(D)** High-throughput sequencing of RNA isolated by crosslinking and immunoprecipitation. **(E)** Photoactivatable-ribonucleoside- enhanced crosslinking and immunoprecipitation.

Different strategies have been used to isolate the population of mRNAs bound by an RBP, which differ in complexity and in the ability to identify true interactions. Classical RNA Pull-Down approaches (Einarson et al., 2007) involve the use of recombinant-tagged proteins that are immobilized onto different types of supports and purify mRNA in an *in vitro* affinity chromatography assay (Figure 1A).

The tandem affinity purification (TAP-tag) method involves the fusion of a double-tag either at the amino or carboxy terminus of the protein followed by transfection of the studied cell type (Figure 1B). *In vivo*-formed RNA-protein complexes are purified by two-step affinity chromatography with tag-specific binding proteins (Puig et al., 2001; Gerber et al., 2006). However, the presence of the tag could interfere with native interactions, yielding false, or at least incomplete patterns of binding.

RNA targets of RBPs have been identified by immunoprecipitation assays, followed by genomic analysis using microarrays, known as RIP-Chip (RNA ImmunoPrecipitation and microchip hybridization), or more recently using next-generation sequencing methods, RIP-Seq (Figure 1C) (Tenenbaum et al., 2002; Jayaseelan et al., 2011, 2014). These techniques could be considered gold-standard techniques, as we are isolating the native complexes under normal conditions. The effectiveness of this approach relies in the purity and affinity of the antibodies that are used.

One major unwanted output of these techniques is the co-purification of non-specific and usually abundant transcripts. To isolate only specific transcripts that are bound by RBPs, different strategies have been developed to improve the RIP analysis. The refinement of this technique involves the cross-linking of RNA-protein complexes prior to purification (CLIP) (Figure 1D). Ultraviolet light causes the formation of covalent bonds between RNAs and proteins in direct or close contact. CLIP assays reduce the rate of false positives, and when combined with nuclease treatments, they can give precise information about the RNA element that is recognized and bound by RBPs (Ule et al., 2003; Jensen and Darnell, 2008). HITS-CLIP (high-throughput sequencing of RNA isolated by crosslinking immunoprecipitation) or CLIP-Seq represent high-throughput methods that were developed to generate genome-wide RNA–protein interaction maps (Licatalosi et al., 2008).

To map the binding site, RNA is digested up to a length of ~50 nt, reverse transcribed after RNA adapter ligation, and amplified to prior sequencing. One limitation is represented by the low efficiency of crosslinking using UV light. PAR-CLIP (PhotoActivatable Ribonucleoside-enhanced CrossLinking and ImmunoPrecipitation) (Hafner et al., 2010a) has been developed to more precisely map the exact binding sites at the nucleotide resolution and to increase the efficiency of the crosslinking (Figure 1E). PAR-CLIP is based on the incorporation of photoreactive ribonucleoside analogs (4-thiouridine or 6-thioguanosine) into newly synthesized RNAs. The use of ribonucleoside analogs has two advantages: they allow crosslinking with UV light at 365 nm, and they lead to a base transition during reverse transcription (thymidine to cysteine or guanosine to adenosine when using 4-thiouridine or 6-thioguanosine, respectively), which can be used to define the crosslink site at a nucleotide resolution (Hafner et al., 2010b).

These techniques allow the identification of subsets of RNAs that have related functions and are potentially co-regulated (Jayaseelan et al., 2014). In this review, we focus on recent insights from the RNA target of RBPs studies carried out in stem cells and progenitor cells that have contributed to understanding the post-transcriptional regulation of self-renewal and cell differentiation processes.

## Stem cell ribonomics and gene regulatory networks in self-renewal and differentiation

The ribonomic approach has been used to identify the gene regulatory networks that are formed by the RBPs and mRNAs that are involved in the commitment of stem cell differentiation. Nevertheless, other strategies have been used that identify *bona fide* targets of RBPs. Mex3 is a maternal totipotency factor that controls mRNA metabolism in oocytes and germline stem cells (Draper et al., 1996). This protein has a conserved dual KH domain that is involved in the direct binding of the 3′UTR of transcripts (Jadhav et al., 2008). In *C. elegans*, the Mex3 binding site was determined using the SELEX approach (Pagano et al., 2009). The defined Mex3 recognition element was used to search a *C. elegans* transcript database for putative target mRNAs. Hits were filtered to include only transcripts that are expressed in embryonic stem cells identifying 214 candidate Mex3 targets. A detailed analysis of these targets showed the presence of multiple regulatory RBPs, such as members of the PUF family, GLD1 and Nanos2, and essential pluripotency factors, such as SOX2. These results allowed the authors to understand the dual role of Mex3 in regulating germline stem cell totipotency and embryonic cell fate specification.

The RIP assay has also been combined with other techniques, not as powerful as high-throughput sequencing that allowed the identification of mRNA targets. The DEAD END (DND1) protein is essential for the maintenance of viable stem cells. This protein is expressed in human embryonic stem cells, where the population of associated mRNAs was identified (Zhu et al., 2011). The strategy was to overexpress a hemagglutinin (HA)-tagged protein in human embryonic stem cells. Bound transcripts were identified by RIP, followed by RT-PCR. Transcripts encoding pluripotency factors, such as Oct4, Sox2, and Nanog, were identified as associated with the protein. This approach showed that DND1 is a post-transcriptional regulator of the expression of pluripotency factors that are essential for stem cell maintenance.

The identification of target mRNAs showed new functions of RBPs that were not evident or were shaded by a dominant phenotype in reverse genetic assays. There are several examples where the identification of RBP-associated transcripts led to the discovery of essential pathways in stem cell fate regulation.

A deficiency in the fragile X mental retardation protein (FMRP) is responsible for fragile X syndrome. FMRP contains multiple domains that directly interact with RNA (Li and Zhao, 2014). FMRP regulates mRNA expression by repressing translation (Napoli et al., 2008) and by interacting with the miRNA machinery (Caudy et al., 2002; Ishizuka et al., 2002). FMRP is essential for the maintenance of germ stem cells and adult and embryonic stem cells (Li and Zhao, 2014). In Drosophila germ stem cells, RIP assays were used to purify small RNAs that were bound by FMRP. TaqMan assays were then used to identify 72 different miRNAs (Yang et al., 2009). The authors found that the bantam miRNA was responsible, along with FMRP, for the maintenance of the germ stem cell population. In mouse adult neural stem cells, FMRP regulates genes that are critical for stem cell function (Luo et al., 2010). RIP-Chip assays showed the presence of genes that are classified as enhancers of cell cycle progression and Wnt signaling. Genetic analysis confirmed the role of FMRP in the control of cell specification and Wnt signaling. Moreover, an analysis of the regulatory pathways that are controlled by FMRP suggests that this protein plays an important role in learning and memory formation.

Identifying associated mRNAs can also suggest the mechanisms of gene regulation of an RBP. In neural progenitor stem cells, Boris association with mRNA was analyzed by native RIP and hybridization to Affymetrix gene expression arrays (Ogunkolade et al., 2013). Transcripts corresponding to the ribosomal RNAs (rRNAs) 18S and 28S were overrepresented, suggesting the association of Boris with translating ribosomes, which was confirmed by the western blotting of polysomal fractions. Genes that are involved in the Wnt and cadherin signaling pathways were overrepresented, as well as many genes that are classified as encoding RBPs. These results were confirmed, as the overexpression of Boris leads to the activation of the Wnt canonical pathway. Hence, not only were the putative targets of Boris identified, but the presence of rRNAs suggests that Boris is a translational regulator that exerts its function through association with translating polysomes.

Combining ribonomic results with cell transcriptomics could also define a protein's function. ZFP36l2 is an RBP that is involved in the self-renewal of mouse hematopoietic stem cells. Associated mRNAs that were identified by RIP-Chip showed the presence of AU-rich motifs in their 3′UTR. These mRNAs are induced or preferentially expressed during erythroid differentiation. ZFP36l2 expression is negatively correlated with the expression pattern of its target transcripts, strongly suggesting that it is a negative regulator at the post-transcriptional level (Zhang et al., 2013).

The emergence of large-scale sequencing rendered a new powerful tool to identify not only already known transcripts but also unannotated and non-coding RNAs. RbFox2 is involved in linking non-sense-mediated decay (NMD) mechanisms with alternative splicing regulation. In mouse embryonic stem cells, a stringent purification method was used when iCLIP libraries were generated by sequential Flag and HA tag immunoprecipitation (Jangi et al., 2014). Through RNA-Seq analysis, the authors were able to identify hundreds of splicing events that were associated with RbFox2 RNA binding to intron regions. Moreover, an unexpected enrichment in genes that are regulated by AS-NMD, particularly RBPs, was observed. This observation, that hundreds of silent splicing events bound by Rbfox2 are putative AS-NMD cassette exons suggests that functional splicing regulatory activity can be attributed to the majority of Rbfox2-binding events.

Specific functions of large multifunctional protein complexes can also be dissected using the ribonomic approach. Polycomb proteins play essential roles in stem cell renewal. Antibodies against the EZH2 subunit, which interacts directly with RNA, were used for native RIP-Seq in mouse eukaryotic stem cells (Zhao et al., 2010). A total of 9788 transcripts were found as putative targets, including poly-adenylated transcripts, non-coding RNAs and unannotated sequences (Lee, 2010). The characterization of a polycomb “transcriptome” suggests that the existence of RNA cofactors may be a general feature of polycomb regulation. The ribonomic approach could be useful to identify RNA cofactors for other chromatin modifiers.

Another example of how characterizing RNA-protein interactions could help to understand the biological role of an RBP in stem cells is the Musashi family of proteins (MSI). The Musashi proteins are found in stem and progenitor cells and are overexpressed in several cancer cells, with a well-defined role in the regulation of the undifferentiated state. A pulldown assay (RNA bind and seq) using RNA from mouse neural stem cells identified the population of MSI1-bound transcripts (Katz et al., 2014). The MSI1 protein was fused to a streptavidin-binding peptide, and RNA was pulled down and sequenced under high throughput. This strategy defined a binding element that was present in the 3′UTR of mRNAs. Regarding MSI2, ribonomic assays were performed in hematopoietic stem cells. Park et al. (2014) overexpressed a flag-tagged MSI2 protein and used HITS-CLIP to identify 1097 putative targets of the protein. Among the subpopulation of mRNAs were RNA fate regulators and genes that are involved in the regulation of cell signaling and developmental pathways (Park et al., 2014). The gene network that was defined by the target genes showed that MSI2 is involved in stem cell self-renewal and TGFβ signaling.

## The PUF (pumilio/FBF1) family of RNA-binding proteins and the control of stem cell proliferation

PUF (Pumilio and FBF) proteins are mRNA regulators with a conserved role in the maintenance of mitotic division, resulting in the self-renewal of stem cells (Wickens et al., 2002). Putative PUF target mRNAs have been identified on a genomic scale in budding yeast, human HeLa cells, human adipose-derived stem cells and fly ovaries and embryos (Gerber et al., 2004, 2006; Galgano et al., 2008; Morris et al., 2008; Shigunov et al., 2012). The composition of the associated population of mRNAs depends on the cell transcriptome (Shigunov et al., 2012; Abil et al., 2014). Nevertheless, a comparison of putative FBF and PUF targets in metazoans revealed 40 common transcripts, including well-established stem cell fate regulators (Kershner and Kimble, 2010).

PUF proteins were immunoprecipitated with bound mRNAs, and those RNAs were then used to probe microarrays (RIP-Chip) (Galgano et al., 2008; Kershner and Kimble, 2010; Shigunov et al., 2012). The FBF target mRNAs represent ~7% of the *C. elegans* protein-coding genes, and PUF proteins in humans and Drosophila likely control a similar proportion (7–11%) of their respective transcriptomes (Gerber et al., 2006; Galgano et al., 2008; Morris et al., 2008; Kershner and Kimble, 2010). Putative PUM1 and PUM2 targets have been analyzed in HeLa and HEK293 cells, showing that these proteins bind a large set of transcripts with a large overlap of putative targets (Galgano et al., 2008; Morris et al., 2008; Hafner et al., 2010b). One of the common biological processes of PUF target mRNAs is cell proliferation control, which has been demonstrated by several groups (Kennedy et al., 1997; Crittenden et al., 2002; Lee et al., 2007; Ariz et al., 2009; Kalchhauser et al., 2011; Chen et al., 2012; Racher and Hansen, 2012; Van Etten et al., 2012).

**Figure 2:**
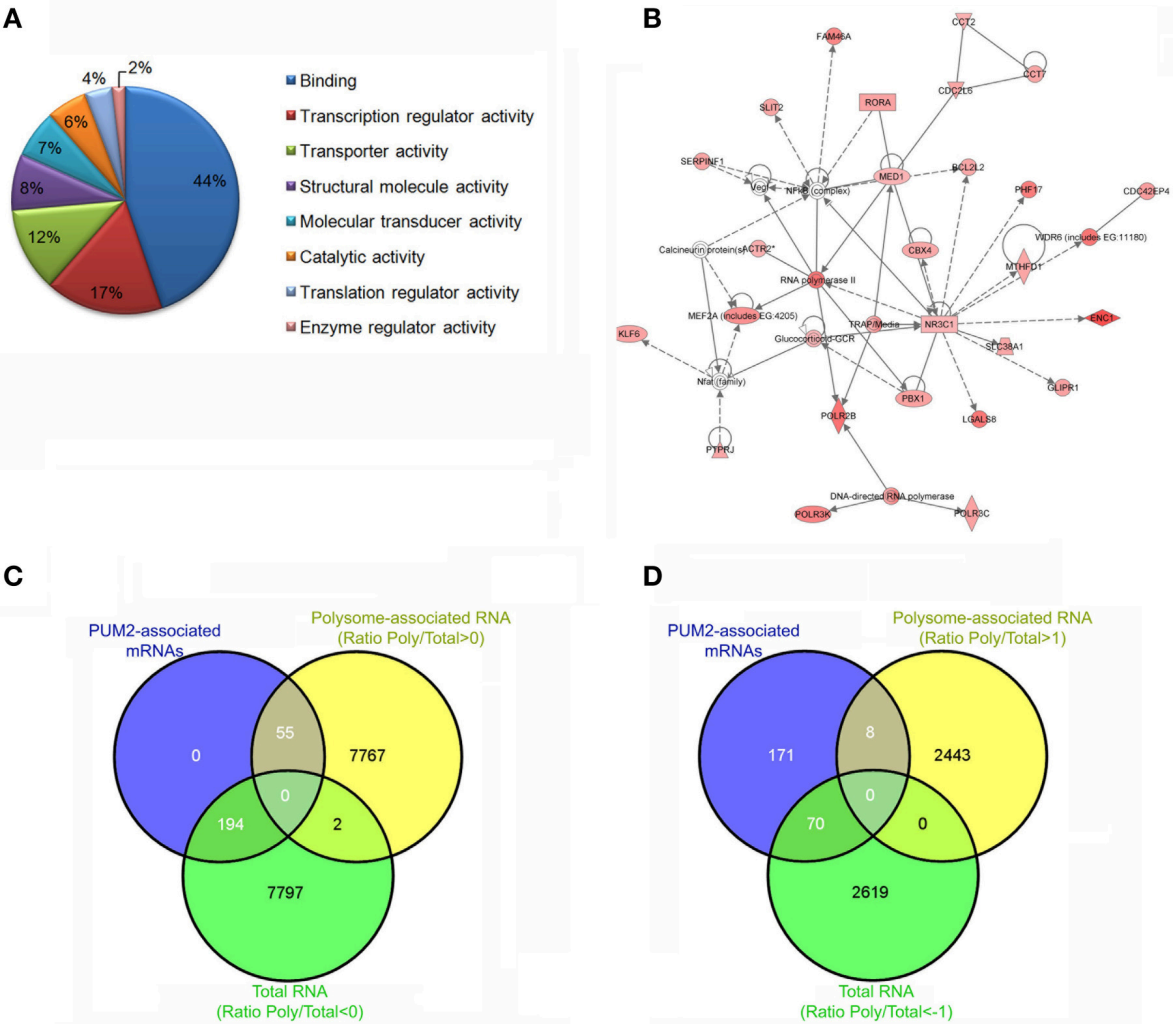
**Steps to Ribonomic analysis of PUM2-associated mRNAs**. Ensure that the protein of interest was specifically immunoprecipitated using western blotting. Identify and cluster the mRNAs according to their enrichment and the biological and technical replicates. **(A)** Cluster the mRNAs according to biological process, cellular component, and molecular function. **(B)** Identify networks of mRNAs that are associated with PUM2. **(C)** Comparison of the PUM2-associated mRNAs with Total RNA (ratio < 0) and polysome-associated mRNAs (ratio > 0). Ratio polysome-associated RNAs/Total RNA. **(D)** Comparison of the PUM2-associated mRNAs with Total RNA and polysome-associated mRNAs enriched < 1 and >1, respectively.

Our group was able to identify approximately 300 putative PUM2 targets in adipose-derived stem cells using RIP-Chip (Figures 2A,B) (Shigunov et al., 2012). Cellular growth and proliferation were associated as network functions of putative PUM2 targets by a highest score analysis. Among the putative PUM2 targets, we also found several transcripts encoding proteins that are involved in the negative regulation of proliferation. Several transcripts encoding cell cycle-related proteins were found to be associated with PUF proteins in different organisms, suggesting that PUF proteins could be directly involved in the control of progression through the cell cycle (Galgano et al., 2008; Morris et al., 2008; Hafner et al., 2010b; Kershner and Kimble, 2010).

In another study from our group, we evaluated the total RNAs and polysome-associated mRNAs in adipose-derived stem cells using polysome profiling (Spangenberg et al., 2013). We reanalyzed the data (ArrayExpress E-MTAB-1366) to identify RNAs that were enriched in the polysomal fraction by calculating the ratio between polysome-associated and total RNA. When we compared the previously described PUM2-associated mRNAs with total RNAs and polysome-associated mRNAs (Oliveros, 2007), we found only 22% of PUM2-associated mRNAs in the polysomal fraction (Figure 2C). If we look at the elements that are enriched by fold change (1 > log < −1), this number decreases to 10% (Figure 2D). Pumilio represses the translation of specific mRNAs by recruiting factors that control RNA stability (Goldstrohm et al., 2006), and Pum2 represses translation by competing with eIF4E to bind the cap (Cao et al., 2010). Our results suggest that PUM2 could prevent the mRNAs from associating with ribosomes by competing with eIF4E. PUMILIO-1 and 2 RBPs have been previously identified as regulators that are involved in stem cell proliferation in invertebrates (Catelain et al., 2014). PUF proteins in concert with other proteins coordinate the temporal or spatial pattern of translation of a large set of mRNAs. Experiments to identify tissue-specific mRNA targets of PUF will also allow the determination of the landscape of mRNAs and partners that are unique to each cell type, contributing to the understanding of protein function.

## Conclusion

The identification of which RNAs are associated with post-transcriptional regulatory proteins and the genetic network that they form is important to understand the essential steps in stem cell differentiation and the mechanisms that are responsible for the maintenance of the undifferentiated state. The combination of affinity purification methods and large-scale identification of transcripts, plus the development of powerful bioinformatics tools will lead to a systematic characterization of these networks. Though much effort has been devoted to this challenge, there is still much work in the future that is needed to understand the complex mechanisms underlying post-transcriptional regulation.

## Author contributions

The authors contributed equally to write the mini review.

### Conflict of interest statement

The authors declare that the research was conducted in the absence of any commercial or financial relationships that could be construed as a potential conflict of interest.
